# The Nomogram predicting the overall survival of patients with pancreatic cancer treated with radiotherapy: a study based on the SEER database and a Chinese cohort

**DOI:** 10.3389/fendo.2023.1266318

**Published:** 2023-10-25

**Authors:** Xiaotao Dong, Kunlun Wang, Hui Yang, Ruilan Cheng, Yan Li, Yanqi Hou, Jiali Chang, Ling Yuan

**Affiliations:** ^1^ Department of Radiation Oncology, Affiliated Cancer Hospital of Zhengzhou University, Zhengzhou, Henan, China; ^2^ Department of Hematology and Oncology, Shenzhen Children’s Hospital Affiliated to China Medical University, Shenzhen, China

**Keywords:** pancreatic cancer, radiotherapy, SEER database, LASSO regression, nomogram

## Abstract

**Objective:**

Patients with pancreatic cancer (PC) have a poor prognosis. Radiotherapy (RT) is a standard palliative treatment in clinical practice, and there is no effective clinical prediction model to predict the prognosis of PC patients receiving radiotherapy. This study aimed to analyze PC’s clinical characteristics, find the factors affecting PC patients’ prognosis, and construct a visual Nomogram to predict overall survival (OS).

**Methods:**

SEER*Stat software was used to collect clinical data from the Surveillance, Epidemiology, and End Results (SEER) database of 3570 patients treated with RT. At the same time, the relevant clinical data of 115 patients were collected from the Affiliated Cancer Hospital of Zhengzhou University. The SEER database data were randomly divided into the training and internal validation cohorts in a 7:3 ratio, with all patients at The Affiliated Cancer Hospital of Zhengzhou University as the external validation cohort. The lasso regression was used to screen the relevant variables. All non-zero variables were included in the multivariate analysis. Multivariate Cox proportional risk regression analysis was used to determine the independent prognostic factors. The Kaplan-Meier(K-M) method was used to plot the survival curves for different treatments (surgery, RT, chemotherapy, and combination therapy) and calculate the median OS. The Nomogram was constructed to predict the survival rates at 1, 3, and 5 years, and the time-dependent receiver operating characteristic curves (ROC) were plotted with the calculated curves. Calculate the area under the curve (AUC), the Bootstrap method was used to plot the calibration curve, and the clinical efficacy of the prediction model was evaluated using decision curve analysis (DCA).

**Results:**

The median OS was 25.0, 18.0, 11.0, and 4.0 months in the surgery combined with chemoradiotherapy (SCRT), surgery combined with radiotherapy, chemoradiotherapy (CRT), and RT alone cohorts, respectively. Multivariate Cox regression analysis showed that age, N stage, M stage, chemotherapy, surgery, lymph node surgery, and Grade were independent prognostic factors for patients. Nomogram models were constructed to predict patients’ OS. 1-, 3-, and 5-year Time-dependent ROC curves were plotted, and AUC values were calculated. The results suggested that the AUCs were 0.77, 0.79, and 0.79 for the training cohort, 0.79, 0.82, and 0.81 for the internal validation cohort, and 0.73, 0.93, and 0.88 for the external validation cohort. The calibration curves Show that the model prediction probability is in high agreement with the actual observation probability, and the DCA curve shows a high net return.

**Conclusion:**

SCRT significantly improves the OS of PC patients. We developed and validated a Nomogram to predict the OS of PC patients receiving RT.

## 1 Introduction

Pancreatic cancer (PC) is highly malignant with a poor prognosis. According to statistics, PC is the seventh leading cause of cancer death, with a 5-year survival rate of less than 10% (1), and the incidence of PC is gradually increasing in developing and developed countries (2). Surgery is the first choice for the treatment of PC. Due to the late diagnosis of PC, more than 80% of PC patients are unresectable at the time of diagnosis, and unresectable PC patients usually follow the basic principles of chemoradiotherapy (CRT)-based, multidisciplinary, and comprehensive treatment (3). With the current development of Computer Science and Technology, Physics, and imaging technologies [e.g., Cyberknife, gamma knife, MRI localization guidance (4)], the occurrence of acute gastrointestinal toxic reactions due to radiotherapy(RT) is significantly reduced. Stereotactic Body Radiation Therapy (SBRT) for treating PC can obtain a high local tumor control rate and significant clinical benefits (5). Currently, neoadjuvant CRT for critically resectable PC requires the following conditions: tumor diameter ≤6 cm; no lymph node metastasis; ≥1 cm from the gastrointestinal mucosa ; and no obvious manifestation of tumor invasion (6–10). The safety of the neoadjuvant FOLFIRINOX chemotherapy regimen combined with SBRT is high, with final surgical resection rates ranging from 10% to 21% (11, 12). RT is essential in both the preoperative conversion of PC and the late palliative treatment of unresectable PC. Based on the SEER database and Chinese cohort, we explored the survival and prognostic factors of PC patients treated with RT. We established and validated a Nomogram to predict the OS of PC patients.

## 2 Patients and methods

### 2.1 Data source and data extraction

**Figure 1:**
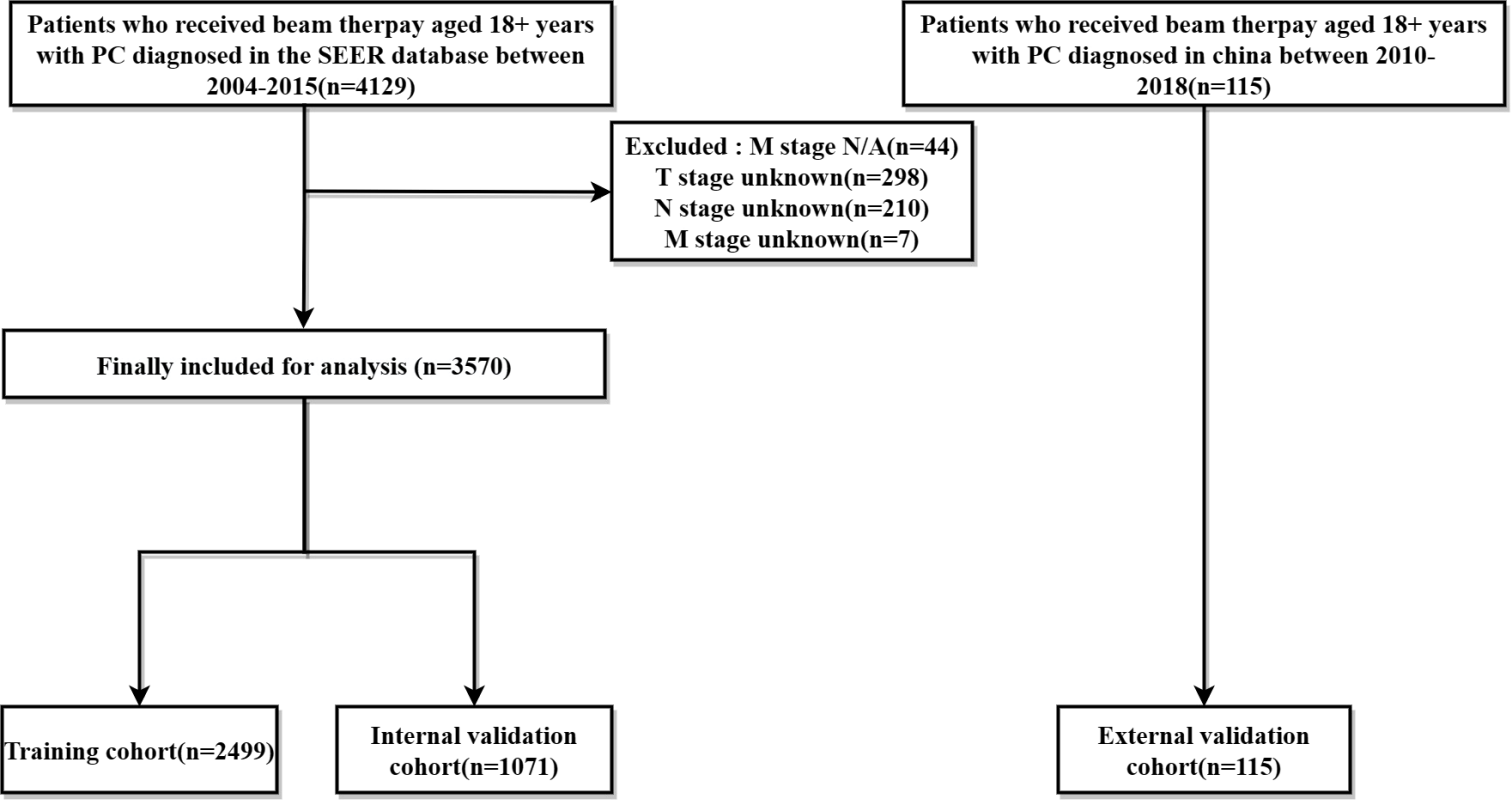
Flow chart of the study. PC: pancreatic cancer.

Data on PC patients receiving RT were collected from the SEER database and The Affiliated Cancer Hospital of Zhengzhou University, respectively. The SEER*Stat software (version 8.4.0) produced by the Surveillance Research Program and National Cancer Institute was applied to identify PC patients undergoing beam therapy. Inclusion criteria were as follows: (1) patients were 18+ years of age; (2) pathological diagnosis of single primary PC; (3) precise pathological diagnosis; (4) treated with external irradiation (Beam radiation), exclusion criteria include: (1) multiple primary cancers; (2) patients with missing information related to radiotherapy; (3) missing survival data, T, N, M stage were not clear; Finally, The SEER database of 3570 cases and The Affiliated Cancer Hospital of Zhengzhou University of 115 PC patients from were included in this study. The ethics committee reviewed and approved this study involving human participants at The Affiliated Cancer Hospital of Zhengzhou University, Zhengzhou, China. Written informed consent from the patients/participants was not required to participate in this study in accordance with the national legislation and institutional requirements. The screening flow chart is shown in (**Figure 1**).

### 2.2 Data classification

The variables included in the study included age, gender, race, marriage, primary tumor site, tumor size, histological grade, surgery, chemotherapy, T stage, N stage, M stage, and lymph node surgery (1) age was divided into three groups: ≤60 years, (60,70], and >70 years; (2) gender was divided into male and female; (3) race included white, black, and others (including yellow, Indian, etc.) three groups. (4) Marriage was divided into married, unmarried, and others (including widowed and divorced); (5) Primary site was divided into the head of the pancreas, the body of pancreas, the tail of pancreas, and others (including overlapping); (6) Tumor size was divided into ≤3cm; 3-5cm; 5-10cm; >10cm and others; (7) Histological grade was divided into grade I, grade II, grade III, grade IV and unknown; (8) The group was divided into surgical and non-surgical groups; (9) The group was divided into chemotherapy and non-chemotherapy groups according to the chemotherapy status. (10) T-stage was divided into T0, T1, T2, T3, T4; (11) N stage was divided into N0, N1; (12) M stage was divided into M0, M1; (13) Lymph node surgery was divided into no surgery, lymph node dissection and lymph node biopsy; the criteria of T stage, N stage, and M stage were referred to the 6th edition of the American Joint Committee on Cancer (AJCC) TNM staging.

### 2.3 Statistical methods

The information of 3570 patients was extracted from the SEER database and analyzed and plotted using R software (version 4.3.0) and GraphPad Prism 8.0. The SEER database data were divided into a training cohort and an internal validation cohort in the ratio of 7:3 by applying the “caret” package. The 115 PC patients from The Affiliated Cancer Hospital of Zhengzhou University were used as the external validation cohort, and the count data were expressed as the number of cases and rate (%). Variables in the modeling group included age, gender, race, marriage, primary tumor site, tumor size, histological grade, surgery status, chemotherapy status, T stage, N stage, M stage, and lymph node surgery. The Kaplan-Meier(K-M) method plotted survival curves for different treatments. The “glmmet” package was used to perform a lasso regression analysis to downscale the variables and screen the best predictor variables, and the best predictor variables were included in the Multivariate Cox proportional risk regression analysis for further screening; at the same time, forest plots were drawn to visualize the HR values; the “rms” package was used to build up a Nomogram based on the results of lasso regression analysis and Multivariate Cox proportional risk regression analysis. The “PROC,” “timeROC,” and “dcurves” packages were used to plot the time-dependent receiver operating characteristic (TROC) curves of the training and validation cohorts of the line graph model to verify the model differentiation. The area under the curve (AUC) was calculated; calibration curves were plotted using Bootstrap with 1000 repetitions of playback sampling; decision curve analysis (DCA) was performed using the “ggDCA” package to evaluate the clinical effectiveness of the line graph model. p<0.05 was considered statistically significant.

## 3 Result

### 3.1 Clinical feature

The baseline characteristics of all enrolled patients were as follows (**Table 1**).

**Table 1:** Comparison of clinical characteristic.

	SEER database	Chinese cohort
	N=3570	N=115
Marriage (%)		
Single	400 (11.2)	12 (10.4)
Married	2299 (64.4)	72 (62.6)
Unknown	871 (24.4)	31 (27.0)
Age (%)		
≤60	1213 (34.0)	41 (35.7)
(60,70]	1234 (34.6)	34 (29.6)
>70	1123 (31.5)	40 (34.8)
Sex (%)		
Female	1738 (48.7)	46 (40.0)
Male	1832 (51.3)	69 (60.0)
Site (%)		
head	2441 (68.4)	87 (75.7)
body	422 (11.8)	7 (6.1)
tail	253 (7.1)	7 (6.1)
others	454 (12.7)	14 (12.2)
Grade (%)		
I	211 (5.9)	4 (3.5)
II	878 (24.6)	29 (25.2)
III	693 (19.4)	21 (18.3)
IV	44 (1.2)	2 (1.7)
Unknown	1744 (48.9)	59 (51.3)
Surgery (%)		
NO/Unknown	2129 (59.6)	69 (60.0)
Yes	1441 (40.4)	46 (40.0)
T (%)		
T0	9 (0.3)	1 (0.9)
T1	104 (2.9)	1 (0.9)
T2	508 (14.2)	20 (17.4)
T3	1974 (55.3)	66 (57.4)
T4	975 (27.3)	27 (23.5)
N (%)		
N0	1893 (53.0)	63 (54.8)
N1	1677 (47.0)	52 (45.2)
M (%)		
M0	3098 (86.8)	105 (91.3)
M1	472 (13.2)	10 (8.7)
Chemotherapy (%)		
NO/Unknown	343 (9.6)	9 (7.8)
Yes	3227 (90.4)	106 (92.2)
Size (%)		
Unknown	1223 (34.3)	41 (35.7)
≤3cm	1592 (44.6)	50 (43.5)
(3,5]cm	527 (14.8)	20 (17.4)
(5,10]cm	24 (0.7)	0(0)
>10cm	204 (5.7)	4 (3.5)
Ln.sug (%)		
NO/Unknown	1962 (55.0)	62 (53.9)
Yes	1495 (41.9)	49 (42.6)
biopsy	113 (3.2)	4 (3.5)
Race (%)		
Black	292 (8.2)	N/A
White	2817 (78.9)	N/A
Others	461 (12.9)	115

Ln.sug, Lymph node surgery; T, T stage; N, N stage; M, M stage.

### 3.2 Subgroup analysis

**Figure 2:**
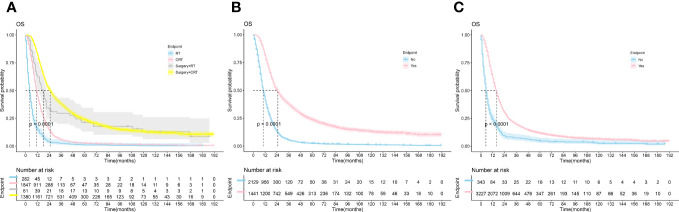
The Kaplan-Meier survival analysis curves for OS according to treatments. **(A)** RT ± Surgery ± Chemotherapy **(B)**: Surgery **(C)**: Chemotherapy; RT, radiotherapy; CRT, chemotherapy combine with radiotherapy.

K-M survival curves were plotted by R studio (**Figure 2**) according to treatment status and were divided into Surgery combined with CRT group (n=1380), Surgery combined with RT group(n=61), CRT group(n=1847), and RT alone group (n=282), with median OS of 25.0, 18.0, 11.0, and 4.0 months, respectively; surgery group (n=1441) and non-surgery group (n= 2129) median OS (25.0 vs. 10.0, P < 0.001); median OS (16.0 vs. 5.0, P < 0.001) in the chemotherapy group (n=3227) and the non-chemotherapy group (n=343), although the specific dosing regimen and treatment sequence in the SCRT group were uncertain, it survival benefit for PC patients could be clarified. Follow-up studies are still needed to determine the optimal treatment modality.

### 3.3 LASSO regression and multivariate Cox regression analysis

**Figure 3:**
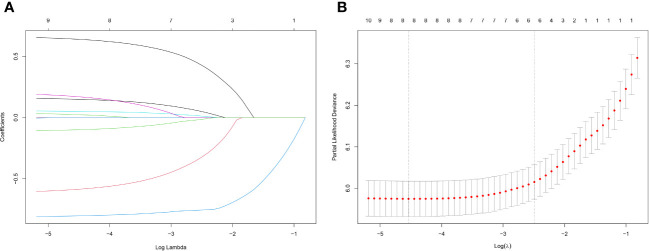
**(A)** Lasso regression variable coefficient versus λ curve; **(B)** Binomial deviation and Lasso regression log(λ) curve, the left vertical dashed line indicates the λ value when the mean square error is smallest, and the right vertical dashed line indicates the λ value when it is one standard error away from the minimum deviation.

**Figure 4:**
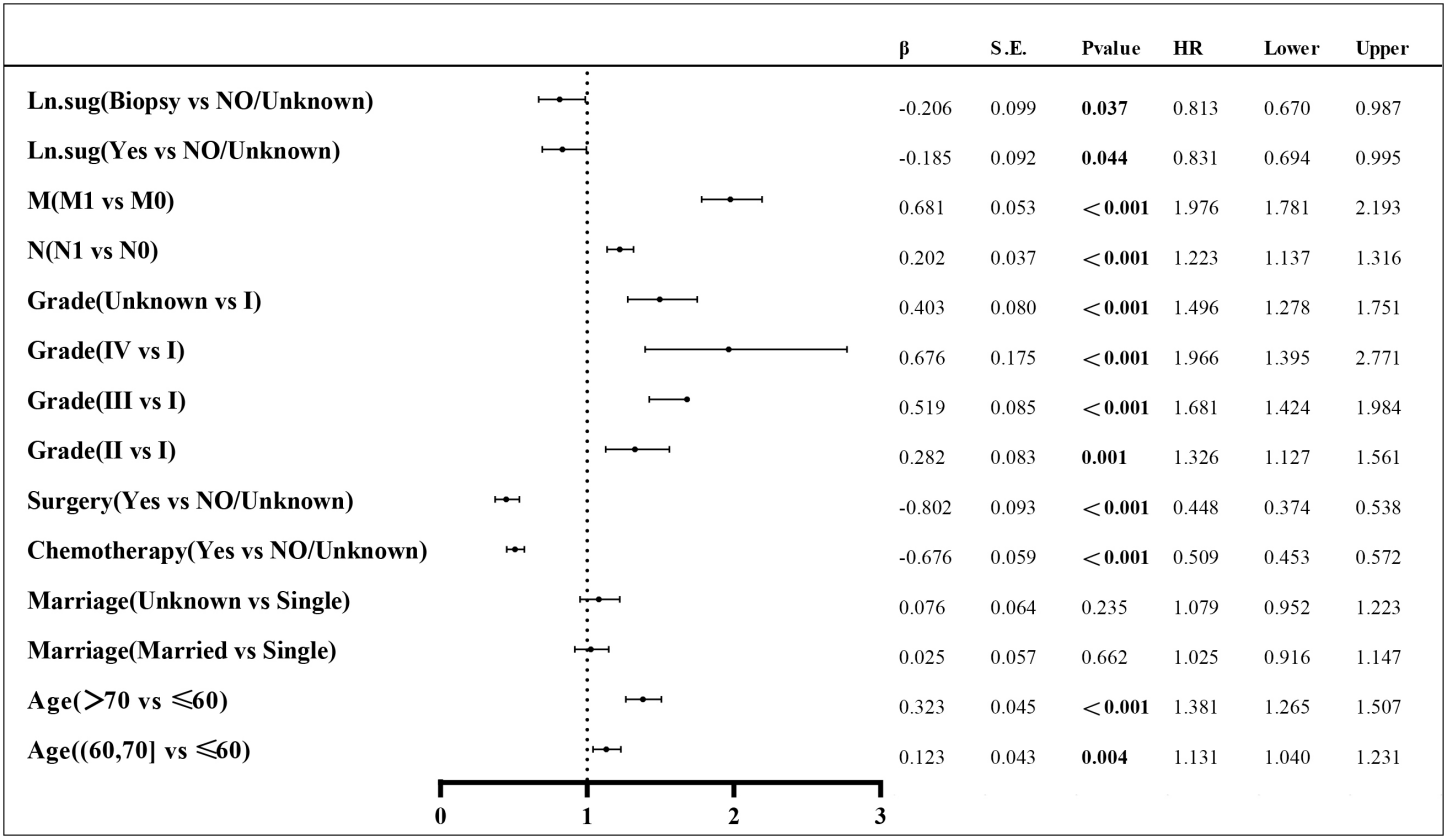
Multivariate Cox regression results; Ln.sug, Lymph node surgery; N, N stage; M, M stage.

Lasso regression is a regression method that limits the complexity of the model by adding L1 regularization. The weight of some independent variables will be reduced or even wholly reduced to 0. This method can eliminate irrelevant features and achieve the purpose of feature selection, which is usually used when there is a correlation or collinearity between variables. A total of 13 clinical characteristics were included in this study to screen the variables most suitable for constructing the Nomogram. The variables in the training group were subjected to lasso regression analysis (**Figure 3**), and all non-zero coefficients were selected demographic and clinicopathological characteristics (age, marriage, Grade classification, N stage, M stage, chemotherapy, surgery, and lymph node surgery) were included in the multivariate Cox regression analysis. The results showed that: older age The results showed that: older age, low-differentiated tumors, late N and M stages, no chemotherapy, no surgery of the primary tumor, and no lymph node surgery were the independent risk factors for patients (**Figure 4**).

### 3.4 The construction and interpretation of Nomogram

**Figure 5:**
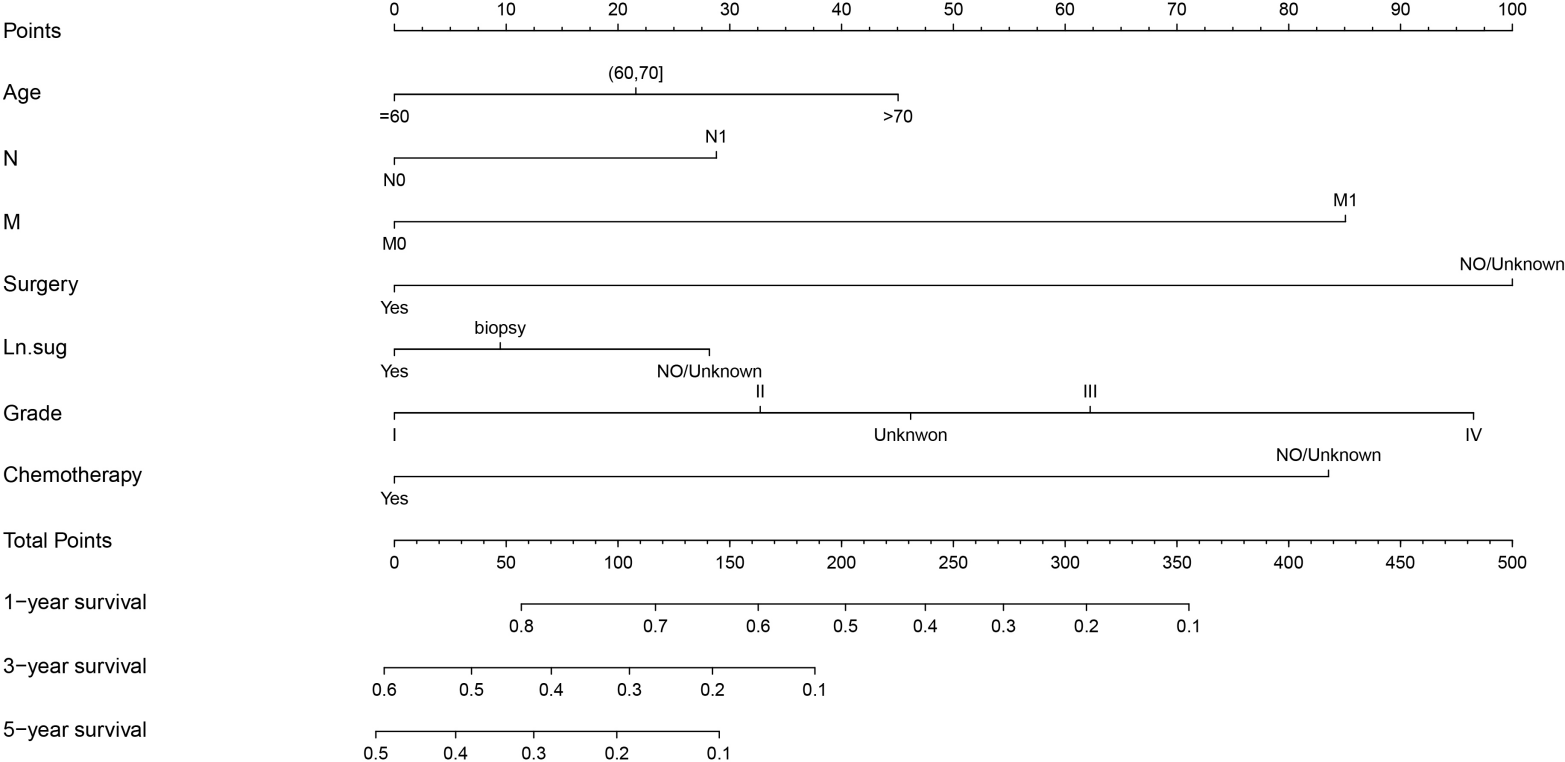
Nomogram for predicting the 1,3,5-year survival in PC with radiotherapy patients. Ln.sug, Lymph node surgery; N, N stage; M, M stage.

The variables with P < 0.05 in the results of the Multivariate Cox regression analysis were included in the Nomogram to construct the prediction model. The scores of each item were obtained by projecting each variable vertically onto the upward scales (Points), and the higher scores were summed to obtain the total scores. The 1, 3, and 5-year survival rates of patients treated with radiotherapy were obtained by projecting the scores vertically from the Total Points to the bottom probability column (**Figure 5**).

### 3.5 The validation of Nomogram and clinical utility

**Figure 6:**
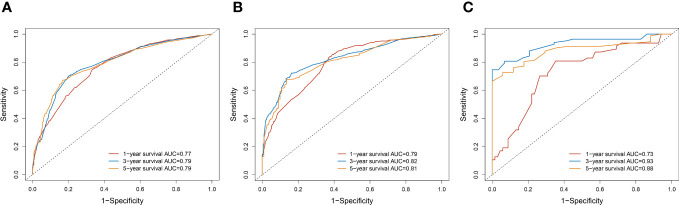
**(A)** TROC curve analysis to predict 1,3,5-year-survival for the Training cohort. **(B)** TROC curve analysis to predict 1,3,5-year-survival for Internal validation cohort. **(C)** TROC curve analysis to predict 1,3,5-year-survival for External validation cohort.

**Figure 7:**
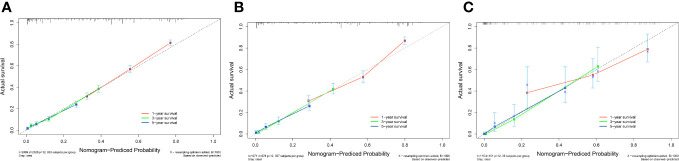
**(A)** Calibration curve of the nomogram for predicting 1,3,5-year survival in PC patients for the Training cohort. **(B)** Calibration curve of the nomogram for predicting 1,3,5-year-survival in PC patients for the Internal validation cohort. **(C)** Calibration curve of the nomogram for predicting 1,3,5-year-survival in PC patients for the External validation cohort. The X-axis represents the probability predicted by the nomogram, and the Y-axis represents the actual probability. Perfect predictions correspond to dashed lines. The red dashed line represents the entire cohort, and the solid line, bias-corrected by Bootstrapping with 1000 replicates, represents the observed performance of the nomogram.

**Figure 8:**
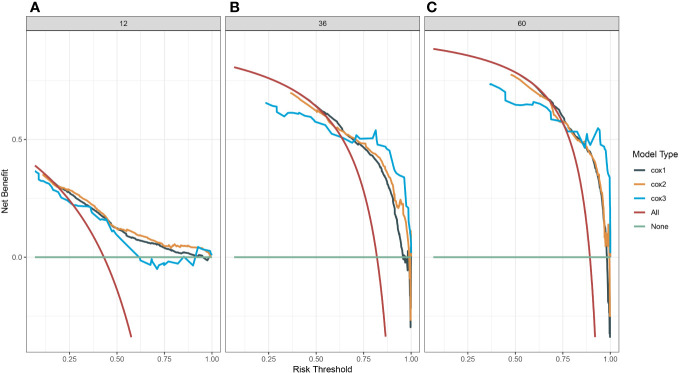
**(A)** Decision clinical curve of the nomogram for predicting 1-year-survival in PC patients. **(B)** Decision clinical curve of the nomogram for predicting 3-year-survival in PC patients. **(C)** Decision clinical curve of the nomogram for predicting 5-year-survival in PC patients. #Cox1, Training cohort; Cox2, Internal validation cohort; Cox3, External validation cohort.

A survival-related Nomogram was constructed based on the results of Multivariate Cox regression analysis, and a cohort of 115 Chinese cases was included as an external validation cohort to plot TROC curves (**Figure 6**), calibration curves (**Figure 7**), and DCA curves (**Figure 8**). The validation of the constructed Nomogram was performed, and the AUC of the training cohort was 0.77, 0.79, and 0.79 for the training cohort, and 0.79, 0.82, and 0.81 for the internal validation cohort, and 0.73, 0.93, and 0.88 for the external validation cohort, respectively. The calibration curves were plotted close to the reference line, indicating that this line plot model predicted 1,3,5-year survival in good agreement with the actual situation. However, it is worth noting that the 3-year TROC curves differed significantly among the three groups. DCA is a method to evaluate prediction models (13), which can evaluate the clinical utility of line plot models by measuring their clinical validity through risk threshold (X-axis) and net benefit (Y-axis). The DCA curve in this study suggested a high net benefit and good clinical prediction.

## 4 Discussion

The prognosis of PC is poor, and most of them are inoperable at first diagnosis. With improving RT techniques, RT is gradually changing from purely palliative to an essential part of multidisciplinary and integrated therapy. To the best of our knowledge, there are few studies on PC patients treated with RT, and developing individualized monitoring and treatment plans is a crucial direction for clinicians. As a novel and simple statistical tool, Nomogram has been widely used in studies related to tumor prognosis (14). The advantage of lasso regression is that it can reduce the dimensionality of multicollinearity predictor variables and filter out the most representative predictor variables, making the model more stable, reducing the model’s complexity, and preventing the overfitting of the model. We applied TROC curves, calibration curves, and DCA to demonstrate the model’s validity and added the Chinese regional cohort as an external validation cohort. The time-dependent ROC is the ROC of sensitivity and 1-specificity calculated for outcomes at different time points. The reasons for the significant differences in TROC at 3-years may be as follows: The sample size of the external validation group was small; The proportion of patients who survived more than three years in the three groups was respectively 457/2499(18.3%),212/1071(19.8%),30/115(26.1%). The highest proportion was found in the external validation cohort. The calibration curves showed that the model had high prediction accuracy, and the DCA showed that the clinical effectiveness of the line graph prediction model was good. All these demonstrate the accuracy, clinical utility, and generalizability of this Nomogram.

Previous studies have shown that age, tumor differentiation, size, serum alkaline phosphatase, albumin, and CA19-9 are independent predictors of prognosis in PC (15). Several prognostic models for PC have been developed. Xu et al. investigated the predictive value of neutrophil/lymphocyte, platelet/lymphocyte, and lymphocyte/monocyte ratios in PC. Shirai (16) et al. showed that the preoperative platelet-to-leukocyte ratio in PC patients can better predict postoperative tumor-free survival and OS. Other serum markers used to construct survival prediction models for PC patients include alkaline phosphatase to albumin ratio (17) and C-reactive protein to serum protein ratio. All these serological markers have been used to predict PC patients’ survival. All these serum markers have been used individually or in combination with other markers to construct survival prognosis models for PC patients, and they have shown good accuracy and sensitivity. In addition, there are also survival prediction models based on PC risk factors, including alcohol consumption, obesity, smoking, and diabetes (18). Moreover, Bioinformatics-based prediction models, including different kinds of miRNAs (19), and models based on patients’ basic clinical information, including age, race, and nutritional status (20, 21).

Based on the SEER database, we constructed a survival prediction model for PC patients treated with radiotherapy. Survival of PC patients treated with radiation therapy and explored the factors affecting their prognosis. Younger are associated with a better survival prognosis and an increased risk of tumor proliferation, metastasis, aging disorders, and immune failure (22). AJCC (TNM) staging is a well-recognized indicator affecting tumor prognosis. There are few studies on the effect of the T stage on OS in tumor patients. Only a study on hepatocellular carcinoma treated with RT in the SEER database found that T1/T2 had a better prognosis than T3/T4 (23). In addition, some studies on metastatic colorectal cancer found that the prognosis of the T0/T1 group was worse than that of the T2/T3 group (24, 25). However, our study did not include the T stage in the Nomogram. Clinicians must clearly understand that early T stage does not mean better OS, and prospective studies are needed to investigate the impact of specific T stage on OS in PC patients. Lymph node metastases and distant metastases mean later N and M stages, usually representing a poorer prognosis (26, 27). A study by Strobel et al. (28)suggests that the number of positive lymph nodes is a preferable predictor of PC prognosis. A retrospective study of stage II operable PC by Morital et al. (29) showed that lymph node metastases were significantly associated with decreased OS. Except for the combination of Oligmetastases in the liver, which can be considered for combined resection depending on the timing, distant metastases at other sites are still contraindicated for surgery. Surgery is an effective treatment to improve OS in patients with PC. Our results showed that the median OS was 25.0 for surgery combined with CRT (**Figure 2**), the same as that of short-course neoadjuvant treatment in a study by Kim et al. (30). As for CRT, Studies have reported the efficacy and adverse effects of SBRT with simultaneous capecitabine, gemcitabine, and 5-fluorouracil. The median OS time was 11.6~14.3 months, and the incidence of grade 3 and above adverse reactions was 7.8%~22.2% (31, 32).

Radiation therapy for PC is controversial regarding the modality of radiation and its combination with drug modalities. The Hypofractionated radiotherapy modality has recently emerged in PC. It is considered superior to the SBRT. Dosimetric studies have shown that adding an intra-target dose on top of the 15-fraction modality is more likely to achieve BED10 = 100 Gy than the 5-fraction modality. Compared with the SBRT(3-5-fraction), Hypofractionated radiotherapy(10-15-fractions) may be the primary modality for PC radiotherapy. However, clinical trial studies still need to be improved in comparing the two modalities. Regarding treatment modalities, In a retrospective study by Zhu et al. (33) comparing the efficacy of RT sequential chemotherapy with induction chemotherapy combined with RT, the median OS of the former was better than that of the latter (13.6m vs. 12.2m), with no significant difference in adverse effects between the two groups. Although RT, chemotherapy, and surgery can all provide survival benefits, the optimal treatment modality remains to be discovered. The Total Neoadjuvant Therapy (TNT)model for PC has been proposed to integrate neoadjuvant RT and adjuvant chemotherapy. Murphy et al. (34, 35) found in two phases II clinical trials using the TNT modality for borderline resectable pancreatic cancer(BRPC) and locally advanced pancreatic cancer (LAPC) that 80% of patients in the BRPC study completed the entire course of treatment and that the R0 resection rate was 96.9% in patients who underwent surgery. OS for all patients was 37.7 months. Disease-free survival (DFS) was significantly improved in surgically resected patients. The 2-year overall survival rate for patients undergoing surgery was 72%. In the LAPC study, approximately 70% of surgical patients achieved R0 resection with the FOLFIRINOX regimen in combination with valsartan treatment. The median DFS and median OS were 17.5 months and 31.4 months for all patients and 21.3 months and 33.0 months for patients who underwent surgery, respectively, with eight cycles of chemotherapy followed by short- or long-course RT, depending on vascular involvement in both studies. In addition, a retrospective study by Truty et al. (36) on BRPC (123 cases) and LAPC (71 cases) also provides a rationale for applying the TNT model to treating PC. With the development of immunotherapy and targeted therapy, combining RT with immunotherapy and targeted agents for treating PC requires prospective clinical studies to identify the optimal combination modality.

There are some limitations of this study. First, this study is a retrospective study based on the SEER database and the Chinese cohort; the model’s applicability to the world is still being determined due to the differences in healthcare systems, disease epidemiology, early screening, and treatment patterns in different countries. Secondly, the study lacks other critical factors associated with PC, such as smoking, alcohol consumption, serological parameters, vascular and lymphatic invasion, inflammatory and specific tumor markers, and essential treatment information, such as particular radiotherapy and chemotherapy regimens and sequences. Then, the lack of CSS data on cancer-related death in this study and the insufficient sample size in the external validation group. Lastly, Patients were enrolled from 2004 to 2015, during which radiotherapy equipment and technique innovations may have affected survival outcomes. The above limitations are also the focus of our future work and the direction of efforts.

The present study is a valuable reference for clinical PC treatment and follow-up studies and a reliable Nomogram model for predicting the OS of PC patients treated with RT, as well as a reference for the design of future multicenter, large sample, and prospective clinical trials.

## Data availability statement

The original contributions presented in the study are included in the article/Supplementary Material. Further inquiries can be directed to the corresponding author.

## Ethics statement

The studies involving humans were approved by The Affiliated Cancer Hospital of Zhengzhou University. The studies were conducted in accordance with the local legislation and institutional requirements. Written informed consent from the patients/participants was not required to participate in this study in accordance with the national legislation and institutional requirements.

## Author contributions

XD: Writing – original draft. KW: Writing – review & editing. HY: Methodology, Writing – review & editing. RC: Investigation, Writing – review & editing. YL: Data curation, Writing – review & editing. JC: Data curation, Investigation, Writing – review & editing. YH: Formal Analysis, Investigation, Writing – review & editing. LY: Supervision, Writing – review & editing.
